# Wind farm and solar park effects on plant–soil carbon cycling: uncertain impacts of changes in ground-level microclimate

**DOI:** 10.1111/gcb.12437

**Published:** 2014-03-28

**Authors:** Alona Armstrong, Susan Waldron, Jeanette Whitaker, Nicholas J Ostle

**Affiliations:** * School of Geographical and Earth Sciences, University of GlasgowGlasgow, UK; † Centre for Ecology and Hydrology, Lancaster Environment Centre, Lancaster UniversityLancaster, UK

**Keywords:** greenhouse gases, land use change, microclimate, solar parks, wind farms

## Abstract

Global energy demand is increasing as greenhouse gas driven climate change progresses, making renewable energy sources critical to future sustainable power provision. Land-based wind and solar electricity generation technologies are rapidly expanding, yet our understanding of their operational effects on biological carbon cycling in hosting ecosystems is limited. Wind turbines and photovoltaic panels can significantly change local ground-level climate by a magnitude that could affect the fundamental plant–soil processes that govern carbon dynamics. We believe that understanding the possible effects of changes in ground-level microclimates on these phenomena is crucial to reducing uncertainty of the true renewable energy carbon cost and to maximize beneficial effects. In this Opinions article, we examine the potential for the microclimatic effects of these land-based renewable energy sources to alter plant–soil carbon cycling, hypothesize likely effects and identify critical knowledge gaps for future carbon research.

## Introduction

This Opinion piece is prompted by our belief that meeting energy demands in a sustainable manner is one, if not the, largest challenge we face today. World primary energy demand is predicted to increase by 40% between 2009 and 2035, with contributions from hydropower, biomass and waste, and ‘other’ renewable energies (primarily wind and solar) predicted to increase by 70%, 55% and 600% respectively (IEA, 2011). From 2010 to 2011 wind power experienced the greatest global GW growth of any renewable technology, bringing total capacity to 238 GW while solar photovoltaic (PV) technology had the highest growth rate (74%) of any renewable energy source, increasing the total capacity to 70 GW (REN21, 2012). Concentrating solar power growth rates were also high (38%) in the same period, but total capacity remains relatively low: 1.8 GW (REN21, 2012). Given the desire for low carbon (C) energy, resource limitations, environmental disasters associated with conventional energy sources such as Fukushima, and the potential of renewable technologies to provide decentralized energy in remote locations, we believe there will be sustained growth of renewable energy technologies in the future. The net result of these changes in energy demand and sources will be an inevitable increase in the establishment of land-based renewables (LBR), solar and wind, energy generation technologies. Solar and wind have the potential to produce energy across the globe, although cost currently restricts the viability in some areas (Pogson *et al*., 2013). The power density of wind and PV are estimated to be 3.0 ± 1.7 and 4–16.5 MW km^−2^ respectively (Denholm & Margolis, 2008; Denholm *et al*., 2009), which using the 2012 global capacities (REN21, 2012), equates to a current land coverage, if all were ground mounted, of 79 000 km^2^ and 4000–17 500 km^2^ for wind and PV respectively. While wind turbines tend to be ground-mounted, PV parks are both building- and ground-mounted, with the relative proportions differing between countries: 45% and 82% of capacity added during 2011 in Europe and China, respectively, were ground mounted (EPIA, 2012). Consequently, hosting LBR represents a substantial global land use change, with the potential to affect plant–soil functions and the supporting (e.g., soil formation, nutrient cycling, primary production), regulating (e.g., climate, disease), provisioning (e.g., food, water) and cultural (e.g., recreation, aesthetics) ecosystem services the landscape provides (Millenium Ecosystem Assessment, 2005).

While there is some understanding of the environmental impacts of LBRs (Smith *et al*., 2011; Pearce-Higgins *et al*., 2012), knowledge of the changes in surface energy fluxes and microclimates is limited, but growing (Baidya Roy *et al*., 2004; Baidya Roy & Traiteur, 2010; Baidya Roy, 2011; Millstein & Menon, 2011; Zhou *et al*., 2012; Adams & Keith, 2013). We argue this knowledge is too incomplete given the rate and potential for LBR deployment. Moreover, there is a considerable knowledge gap on the effects of LBR-altered microclimates on plant and soil processes. Plant–soil interactions govern soil C cycling and storage (Ostle *et al*., 2009a), that underpin critical ecosystem services such as food and timber production, water purification, climate mitigation and nutrient retention (Lal, 2004). Considering the likelihood that land use change for LBR will continue to increase, it is important to ensure that we have scientific understanding of the full impacts on the terrestrial C cycle, greenhouse gas (GHG) emissions and C sequestration. Continuing LBR deployment at the current rate without understanding of ground-level microclimatic effects and the consequent C benefits, or costs, is unwise as we need to ensure any trade-off in the delivery of other ecosystem services is fully considered during planning. Moreover, reducing the embedded C costs in LBR energy production, which could be achieved through increasing soil C sequestration, is one of the key challenges in decarbonizing energy and the future deployment of LBR (Pogson *et al*., 2013): if the effects on C sequestration are positive, the understanding could accelerate our path to sustainable energy provision.

In this Opinions article, we summarize current understanding of LBR-induced changes on microclimates and hypothesize the, as yet unquantified, impacts on plant–soil carbon cycling. We identify and discuss critical knowledge gaps for future carbon research in response to this growing and globally important land use change.

## LBR effects on microclimate

**Fig. 1:**
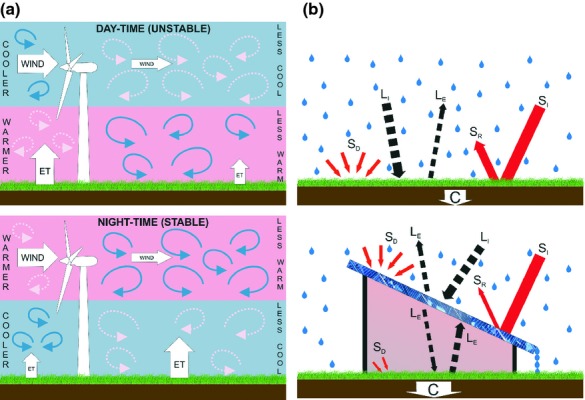
(a) Schematic of the potential effects of wind turbines on air flow, temperature and evapotranspiration during the day with a stable atmospheric boundary layer and at night with an unstable atmospheric boundary layer. The pink (lighter grey) background represents warmer air and blue (darker grey) cooler air. Pink dashed arrows indicate warmer air eddies, which downwind of the turbine are mixed into the cooler air, thus increasing night-time surface temperature. Conversely, the blue solid arrows symbolize cooler air eddies which cause a cooling at the surface during the day-time. The horizontal arrows symbolize the wind speed up and downwind of the turbines, with a reduction in wind speed during the day and night. The vertical arrows suggest hypothesized changes in evapotranspiration, with increases under stable conditions and decreases under unstable conditions downwind of the turbine. (b) Schematic of the potential effects of solar panels on precipitation distribution; incoming shortwave (S_I_), reflected shortwave (S_R_) and diffuse shortwave (S_D_) radiation (solid red arrows); incoming (L_I_) and emitted (L_E_) longwave radiation (dashed black arrows) and conductance (C). The amount of S_R_ will be lower for the photovoltaic (PV) panels, compared with the ground surface, given their lower albedo. The ratio of S_D_ to S_I_ will be greater under the PV as while S_D_ will be reduced nearly all S_I_ will be intercepted by the PV panel. The area under the PV panel is hypothesized to be warmer as a result of L_E_ from the panel, leading to greater conductance into the soil (however, this will be dependent on the effects of the PV panels on wind). Finally, the PV panel will intercept precipitation, concentrating the inputs at the lower edge of the PV panel.

The operation of wind turbines can affect surface meteorology by changing atmospheric boundary layer conditions, namely wind speed, turbulence and mixing, and thus the vertical distribution of energy (heat) and exchange between the land surface and atmosphere (Fig. 1). The installation of ground-mounted PV arrays has the potential to affect surface albedo, cause shading and intercept precipitation and atmospheric deposition, as well as influencing wind speed and turbulence at the land surface (Fig. 1). Local, regional and global effects of wind farms and, to a lesser extent, solar parks on the climate have been postulated (Baidya Roy *et al*., 2004; Keith *et al*., 2004; Millstein & Menon, 2011), with local effects on temperatures within and nearby to wind farms observed (Baidya Roy & Traiteur, 2010; Zhou *et al*., 2012). Changes in wind speed, turbulence and mixing as a result of LBR, may affect humidity (Baidya Roy *et al*., 2004) and potentially biogenic gas [CO_2_, methane (CH_4_) and nitrous oxide (N_2_O)] concentration profiles in the near-surface boundary layer. In addition, large-scale modelling predicts that rainfall could be enhanced by wind farms due to reduced movement of drier air (Fiedler & Bukovsky, 2011), and the LBR-induced changes in temperature and surface heat fluxes could result in a global redistribution of cloud cover and precipitation patterns (Wang & Prinn, 2010). We judge that together all of these phenomena have the potential to interact, causing changes in ground-level microclimatic conditions strong enough to significantly alter plant–soil carbon cycling, with implications for ecosystem and landscape scale GHG emissions and soil C stocks.

## Microclimate effects on plant–soil carbon cycling

**Fig. 2:**
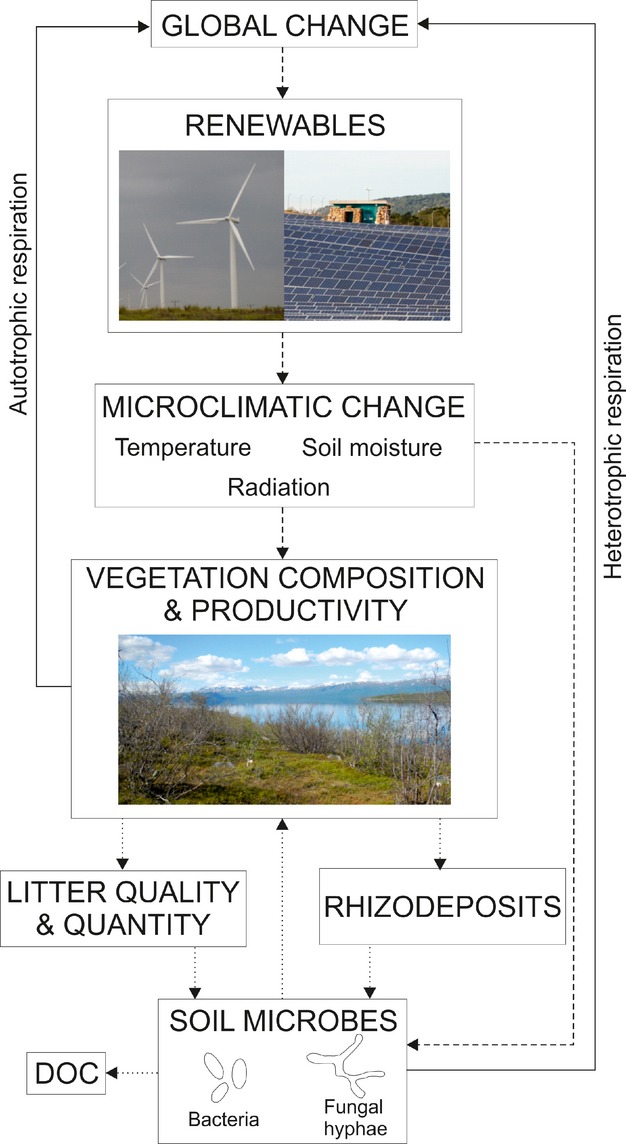
Direct (dashed arrows) and indirect (dotted arrows) effects of LBR-induced changes in microclimate on terrestrial C cycling and feedbacks to global change (solid arrows). Direct effects include the influence of temperature, soil moisture and radiation on plant community composition and productivity, and on soil microbial activity. Indirect effects result from changes in the soil microbial community caused by alteration of soil physico-chemical conditions and C inputs to the soil, mediated through changes in plant community composition and productivity.

Renewable energy generation technologies are being deployed across landscapes with distinct plant–soil communities and C stocks, ranging from C-poor environments (e.g. the Gobi Desert) to C-rich environments (e.g. blanket peatlands of Scotland), in heavily managed (e.g. agricultural land) and relatively unmanaged systems (e.g. deserts). Soil is recognized as the largest single store of terrestrial organic C, containing more C than vegetation and the atmosphere combined (Swift, 2001). Biological plant–soil processes, that interact with biotic and abiotic environmental factors, regulate much of the terrestrial C cycle and thus govern soil C storage, release of greenhouse gas emissions CO_2_, CH_4_ and N_2_O and productivity (Bardgett *et al*., 2008). Climate is a proven powerful determinant of plant–soil processes (Freeman *et al*., 2004; Davidson & Janssens, 2006; Dorrepaal *et al*., 2009; Mercado *et al*., 2009; Allison *et al*., 2010). Consequently, we argue the effects of wind farms and solar parks on the local climate may, therefore, alter the C cycle directly through changes in temperature (air and soil), precipitation and evapotranspiration (and hence soil moisture) and the balance of direct and diffuse radiation (Fig. 2), all of which are proven to influence terrestrial C cycling (Knapp *et al*., 2002; Ma *et al*., 2007; Dorrepaal *et al*., 2009; Mercado *et al*., 2009; Joos *et al*., 2010). However, it is not only the direct effects of the LBR-induced microclimatic change that may alter C cycling, but indirect effects as a result of climate-induced changes in plant and soil microbial community composition and activity (Fig. 2).

In the following sections we summarize the potential effects of microclimatic change caused by LBR on key processes in the terrestrial C cycle and hypothesize the likely implications on productivity, soil C and GHG emissions. We first discuss direct effects, changes in temperature, soil moisture and radiation, on plant–soil C cycling. Then, we discuss indirect effects mediated through changes in plant and microbial communities and describe some of the likely interactive effects.

## Direct effects on plant–soil carbon cycling

### Temperature

Temperature is one of the key drivers of biosphere C cycling, with changes in temperature generally positively related to primary productivity and organic matter decomposition rates, soil DOC concentrations and the uptake and release of CO_2_ and CH_4_ (Clark *et al*., 2009; Dorrepaal *et al*., 2009). However, the direction and magnitude of C response depend on the ecosystem and climatic region (Wise *et al*., 2004; Peng *et al*., 2009). In addition to instantaneous direct effects on plant productivity and decomposition rates, temperature changes caused by LBR may also influence growing season length and consequently ecosystem C cycling through increasing productivity and potentially feedbacks to decomposition given the increased litter inputs and rhizodeposition (Menzel & Fabian, 1999).

The magnitude of measured temperature change caused by wind farms (0.7–3.5 °C (Baidya Roy & Traiteur, 2010; Zhou *et al*., 2012)), and the magnitude of measured warming by solar parks in the built environment (2.5–26.0 °C (Scherba *et al*., 2011); there are no studies of ground-mounted solar parks), are of the order likely to have significant effects on plant productivity and C cycling in various ecosystems. For example, an approximately 1 °C increase in temperature accelerated respiration by 60% in spring and 52% in summer in a subarctic peatland (Dorrepaal *et al*., 2009). Therefore, we are confident that LBR deployment could change productivity and decomposition, but the direction of temperature change is uncertain, that is both increases and decreases in day-time temperature, and increases in night-time temperature have been observed at wind farms (Baidya Roy & Traiteur, 2010; Zhou *et al*., 2012). Increases in day- and night-time temperatures are hypothesized to occur under solar panels in the desert, but day-time decreases could occur if photovoltaic panel technology becomes more efficient (more energy converted into electricity and less emitted as heat) (Millstein & Menon, 2011). Moreover, we postulate that if PV parks are deployed in environments with a lower albedo than deserts, for example grasslands or areas of bare soil, cooling may occur. The relative sensitivity of decomposition and productivity to changes in temperature is debated (Davidson & Janssens, 2006), and therefore we cannot deduce the effect of temperature changes caused by LBR on the C balance of the hosting landscapes with certainty. However, we hypothesize that wind farm-induced increases in night-time temperatures and day-time cooling will accelerate soil decomposition and reduce photosynthesis respectively. Also, we hypothesize that if temperatures increase as a consequence of solar park presence, there will be enhanced soil carbon cycling and GHG emissions. However, the magnitude and direction of ecosystem C response will largely depend on the degree to which the ecosystem is temperature-limited and on the relative importance of other limiting factors including nutrients and soil moisture.

### Soil moisture

Soil moisture, or in wetland soils water table depth, is a dominant abiotic control over productivity and decomposition. While generally productivity and decomposition to CO_2_ will increase with soil moisture there is an upper threshold above which rates decrease, reflecting the response of different plant species to varying soil moistures and the inhibition of decomposition under anaerobic conditions (Sulman *et al*., 2010; Lee *et al*., 2012).

Changes in soil moisture directly affected by LBR are governed by perturbations to both precipitation and evapotranspiration rates. Large-scale wind farms are postulated to affect the distribution of rainfall (Wang & Prinn, 2010) but local effects are not hypothesized. No explicit large-scale effects of solar parks on precipitation are hypothesized, although may occur as solar parks could affect regional temperatures and wind patterns (Millstein & Menon, 2011). However, solar parks will affect the local distribution of precipitation: the areas under the footprint of the panels will receive less, and areas at the edges of the panel will receive more through drainage from the panels (Fig. 1).

The postulated effect of wind turbines on evapotranspiration is small, with an increase in >0.2 mm h^−1^ during stable conditions (Baidya Roy *et al*., 2004), however, as yet there are no published field data supporting this hypothesis. The impact of solar parks on evapotranspiration is less clear and we purport that it will depend on the park design, with potential for increased or decreased rates contingent on whether the surface roughness, and therefore turbulent exchange, is increased or decreased respectively. Therefore, changes in evapotranspiration and precipitation will potentially cause changes in the soil moisture content of soils hosting LBR, but given the limited understanding and paucity of field evidence we cannot conclude the likely direction or magnitude of change. Given the effect of the change in precipitation distribution under and around solar panels, we predict spatially variable soil C concentrations will be promoted. However, we hypothesize that in most LBR hosting ecosystems the effect of soil moisture on plant–soil carbon cycling at the site scale will be relatively minimal given that it is the distribution, not amount, of inputs that will change and preliminary results on evaporation predict small changes.

### Radiation

Solar radiation, and specifically photosynthetically active radiation (PAR), determines the amount of energy available for photosynthesis (Wu *et al*., 2010). Research indicates that diffuse radiation (i.e. scattered) results in enhanced photosynthetic rates (Gu *et al*., 2003) and enhanced soil C sequestration (Mercado *et al*., 2009) compared with direct radiation. The effects of LBR on radiation are, as yet, unknown. We hypothesize that wind farms will have a relatively limited effect on the receipt of PAR, and therefore photosynthesis: there is only short-lived shading from the blades, shading from the turbine tower and a slight increase in the ratio of diffuse to direct radiation due to reflectance of shortwave radiation from the wind turbine. In contrast, we hypothesize solar parks will have substantial effects on the amount of PAR received through interception of a large proportion of the incoming direct and diffuse radiation and that the ratio of diffuse to direct radiation will increase (Fig. 1). We predict the reduction in PAR under the solar panels may cause reductions in photosynthesis and thus productivity. However, in some areas where direct PAR is very high, for example North Africa, photoinhibition and photodamage can occur (Murata *et al*., 2007) making reductions in PAR beneficial to photosynthesis. Consequently, we hypothesize soil C sequestration may increase or decrease, with decreases more likely in regions where low radiation conditions prevail and increases more likely in areas subjected to higher radiation levels.

## Indirect effects on soil carbon cycling

The principal indirect effects of changes in microclimate on plant–soil carbon cycling are a product of longer term changes in plant and soil microbial community composition and shorter term changes in plant carbon inputs. Given the measured and anticipated changes in microclimate we expect, in the longer term, over the 20–25 year lifespan typical of a LBR installation, changes in the vegetation community composition will occur (Euskirchen *et al*., 2009). Given the variability in C cycling between different plant functional types, GHG fluxes and ultimately C sequestration may be altered (De Deyn *et al*., 2008). Indeed, the importance of plant functional type on C cycling has been demonstrated to be greater than climatic effects: litter decomposition rates in one climatic zone were found to vary 18.4-fold, whereas decomposition of the same litter in different climatic zones varied 5.5-fold (Parton *et al*., 2007; Cornwell *et al*., 2008). Change in plant community composition may also lead to other ecological feedbacks that will affect environmental conditions and subsequently soil C cycling. For example, different albedos (Chapin *et al*., 2005) and transpiration rates (Chapin, 2003) are associated with different plant functional types and may affect soil moisture which is a strong C cycle control. Therefore, we advocate consideration of likely changes in vegetation composition in response to the deployment of LBR on terrestrial C cycling.

In the shorter term, we expect direct effects of LBR-induced microclimates on plant productivity may indirectly affect decomposition rates through changes in the quantity and quality of C entering the soil as litter and rhizodeposits (Bardgett *et al*., 2008) (Fig. 2). Additional litter inputs may increase soil C, but can also stimulate increases in soil organic C mineralization and respiration if soil microbes are C limited (Fontaine *et al*., 2004). Litter quality from the same species may change appreciably as a result of changing environmental conditions (Sardans *et al*., 2012), with the quality of litter inputs and rhizodeposits, as determined by plant community composition and abiotic conditions, controlling their decomposability with feedbacks on productivity (Norby *et al*., 2010).

We believe that the research community should also consider the effects of LBR on soil microbial communities. Microbes are a key component of the terrestrial C cycle as they uptake atmospheric CO_2_ and CH_4_ and control the release of these gases through respiration and methanogenesis (Singh *et al*., 2010). Different rates of GHG uptake and release are associated with different microbial groups (Balser & Wixon, 2009), and soil microbial community composition is known to be affected by plant community composition, and abiotic factors (Bardgett *et al*., 2008) Consequently, LBR may affect microbial-mediated GHG emissions and uptake in the short term due to abiotic effects, and in the long term through plant community composition change. Furthermore, changes in microbial communities may feedback and promote further change in plant community composition (Bardgett *et al*., 2008).

As a result of microclimatic-induced changes in respiration and photosynthesis rates, plant and soil communities may affect near-surface CO_2_ concentrations, that feedback and alter plant–soil C cycling rates. LBRs may also affect CO_2_ concentrations directly, through altering mixing and turbulent exchange of near-surface air with the bulk air mass, but we postulate plant–soil effects will dominate. The effect of wind turbines on CO_2_ concentrations has been measured in croplands in central Iowa. Preliminary results indicate higher CO_2_ uptake during the day, more respiration at night but on balance an increase in CO_2_ uptake (see http://www.meteor.iastate.edu/windresearch/researchpapers.html for presentations). Atmospheric CO_2_ concentrations affect plant productivity and decomposition processes, with higher CO_2_ concentrations commonly stimulating higher rates (King *et al*., 2004; Ainsworth & Long, 2005). The balance of assimilation and respiration in response to changes in CO_2_ concentration, and thus the effect on soil C, is variable between studies, but generally elevated CO_2_ increases soil C (Hungate *et al*., 2009).

Thus, there is strong evidence that the combined changes in plant C inputs, plant and microbial community composition and photosynthetic and respiration rates, will act to influence C cycling with feedbacks to GHG emissions. We do not postulate a direction of change as the exact nature of these effects will depend on the ecosystem type (i.e. grasslands, peatlands, deserts, urban environments, rangelands) and local climate, as well as the type and intensity of management (e.g. grazing, cropping, forestry).

## Interactive effects

There is extensive potential for interactive effects between the direct and indirect drivers of the C cycle outlined above, and these may amplify or dampen C cycling processes (Ostle *et al*., 2009b). Interactions studied under climate change scenarios, and we argue others that are specific to LBR-induced ground-level microclimates, are likely to contribute to the regulation of plant–soil C cycling and GHG emissions in landscapes hosting LBR. Many of the common interactions in climate change scenarios have been studied, though less so in relation to effects on microbial communities and CH_4_ fluxes (Singh *et al*., 2010).

Numerous studies have examined the interaction of temperature and soil moisture, two of the dominant variables governing productivity and decomposition. For example, warmer and drier conditions have been associated with increased respiration relative to production across a range of biomes (Anderson-Teixeira *et al*., 2011). Also, the nutrient (e.g., C, N and phosphorous) status of the soil, differences in plant inputs and changes in plant community composition are likely to interact with abiotic drivers to influence C cycling processes. For example, soil C sequestration under elevated CO_2_ is constrained by available N and the nutrients required to support N_2_ fixation (Van Groenigen *et al*., 2006). There is evidence that climate change during the summer months promotes differences in productivity of vascular and nonvascular species (Dorrepaal, 2007), that trees are more responsive than herbaceous species to increases in CO_2_ concentrations (Ainsworth & Long, 2005) and that elevated CO_2_ provides C_3_ plants a competitive advantage over C_4_ plants (Reich *et al*., 2001). There is also evidence that different species, not just different plant functional types, respond uniquely to the same environmental conditions (Dorrepaal, 2007). However, over the lifetime of LBR installations the microclimatic effects on C sequestration may not be as great as hypothesized due to plant acclimation – the change in the biochemical and physiological responses of a plant to environmental change (Luo *et al*., 2001). In addition, there could be larger scale feedbacks on the carbon cycle. For example, warming caused by LBR may increase respiration and thus CO_2_ release, causing a positive feedback and further warming at the global scale. However, this would depend on the scale of LBR deployment globally. These interactions and feedbacks are complex and depend on parameters that are highly variable in time and space; we believe these warrant much scrutiny in further research.

## Future research and conclusions

The speed and scale of land use change associated with the expansion of renewable energy technologies is unprecedented. In our opinion, the challenge for future research is to ensure greater security of energy supply while protecting and potentially enhancing host system terrestrial carbon stocks, productivity and biodiversity. Consequently, we believe that a better scientific understanding of the effects of LBR-induced microclimatic changes on ecosystem carbon cycling and greenhouse gas emissions is critical to allow us to predict and manage impacts and trade-offs across a wide range of hosting landscapes globally. Clearly, the effects of LBR on C cycling rates and plant and soil stocks will be less in ecosystems which, under their current land use, exhibit low rates and stocks, such as deserts and rocky landscapes (we do not advocate or oppose deployment in these environments). The potential to increase C benefits from ground-level changes in microclimate needs to be examined, and placed in the broader context of the full C costs of electricity produced by LBRs; we argue that there is much scope to maximize beneficial effects.

To determine the long-term operational impacts of LBR on plant and soil C, and allow generalization and prediction of effects across the globe, we strongly advocate the investigation of LBR-induced microclimatic effects under different atmospheric conditions, across a range of ecosystems occurring in different climatic zones. Furthermore, understanding and modelling needs to be developed to account for the range of wind farm and solar park designs and consequently designs optimized for energy production and plant–soil C cycling. Therefore, we call for an increase in research effort in this emerging field and propose specific research priorities should be (i) field assessment of the effects of LBR on the local climate, especially solar parks for which there is no evidence, with potential for remote sensing to provide data at a larger scale; (ii) field experiments in carbon relevant hosting ecosystems to examine the effects of LBR-induced microclimates on plant–soil C cycling *in situ*; (iii) controlled environment studies examining the interactive effects of diurnal, seasonal and annual microclimatic controls on plant–soil C cycling; and (iv) modelling that uses mechanistic understanding from field and laboratory studies to upscale, and forecast effects of LBR-induced microclimates on C cycling and greenhouse gas emissions across the globe. Given the dominance of temperature on plant–soil C cycling, it is crucial that new experiments and models examine LBR effects on this parameter. However, the effects of other abiotic and biotic factors that are affected by LBR, and their interactions, also need to be resolved, across the full range of hosting systems.

Land use change for LBR is global, widespread and predicted to increase. Understanding of microclimatic effects is growing, but currently incomplete, and subsequent effects on plant–soil C cycling, GHG emissions and soil C stocks are unknown. We urge the scientific community to embrace this research area and work across disciplines, including plant–soil ecology, terrestrial biogeochemistry and atmospheric science, to ensure we are on the path to truly sustainable energy provision.

## References

[b1] Adams AS, Keith DW (2013). Are global wind power resource estimates overstated?. Environmental Research Letters.

[b2] Ainsworth EA, Long SP (2005). What have we learned from 15 years of free-air CO_2_ enrichment (FACE)? A meta-analytic review of the responses of photosynthesis, canopy properties and plant production to rising CO_2_. New Phytologist.

[b3] Allison SD, Wallenstein MD, Bradford MA (2010). Soil-carbon response to warming dependent on microbial physiology. Nature Geoscience.

[b4] Anderson-Teixeira KJ, Delong JP, Fox AM, Brese DA, Litvak ME (2011). Differential responses of production and respiration to temperature and moisture drive the carbon balance across a climatic gradient in New Mexico. Global Change Biology.

[b5] Baidya Roy S (2011). Simulating impacts of wind farms on local hydrometeorology. Journal of Wind Engineering and Industrial Aerodynamics.

[b6] Baidya Roy S, Traiteur JJ (2010). Impacts of wind farms on surface air temperatures. Proceedings of the National Academy of Sciences.

[b7] Baidya Roy S, Pacala SW, Walko RL (2004). Can large wind farms affect local meteorology?. Journal of Geophysical Research.

[b8] Balser TC, Wixon DL (2009). Investigating biological control over soil carbon temperature sensitivity. Global Change Biology.

[b9] Bardgett RD, Freeman C, Ostle NJ (2008). Microbial contributions to climate change through carbon cycle feedbacks. ISME Journal.

[b10] Chapin FS (2003). Effects of plant traits on ecosystem and regional processes: a conceptual framework for predicting the consequences of global change. Annals of Botany.

[b11] Chapin FS, Sturm M, Serreze MC (2005). Role of land-surface changes in arctic summer warming. Science.

[b12] Clark JM, Ashley D, Wagner M, Chapman PJ, Lane SN, Evans CD, Heathwaite AL (2009). Increased temperature sensitivity of net DOC production from ombrotrophic peat due to water table draw-down. Global Change Biology.

[b13] Cornwell WK, Cornelissen JHC, Amatangelo K (2008). Plant species traits are the predominant control on litter decomposition rates within biomes worldwide. Ecology Letters.

[b14] Davidson EA, Janssens IA (2006). Temperature sensitvity of soil carbon decomposition and feddbacks to climate change. Nature.

[b15] De Deyn GB, Cornelissen JHC, Bardgett RD (2008). Plant functional traits and soil carbon sequestration in contrasting biomes. Ecology Letters.

[b16] Denholm P, Margolis RM (2008). Impacts of Array Configuration on Land-Use Requirements for Large-Scale Photovoltaic Deployment in the United States.

[b17] Denholm P, Hand M, Jackson M, Ong S (2009). Land-Use Requirements of Modern Wind Power Plants in the United States.

[b18] Dorrepaal E (2007). Are plant growth-form-based classifications useful in predicting northern ecosystem carbon cycling feedbacks to climate change?. Journal of Ecology.

[b19] Dorrepaal E, Toet S, Van Logtestijn RSP, Swart E, Van De Weg MJ, Callaghan TV, Aerts R (2009). Carbon respiration from subsurface peat accelerated by climate warming in the subarctic. Nature.

[b20] EPIA (2012). Global Market Outlook for Photovoltaics until 2016.

[b21] Euskirchen ES, Mcguire AD, Chapin FS, Yi S, Thompson CC (2009). Changes in vegetation in northern Alaska under scenarios of climate change, 2003–2100: implications for climate feedbacks. Ecological Applications.

[b22] Fiedler BH, Bukovsky MS (2011). The effect of a giant wind farm on precipitation in a regional climate model. Environmental Research Letters.

[b23] Fontaine S, Bardoux G, Abbadie L, Mariotti A (2004). Carbon input to soil may decrease soil carbon content. Ecology Letters.

[b24] Freeman C, Fenner N, Ostle NJ (2004). Export of dissolved organic carbon from peatlands under elevated carbon dioxide levels. Nature.

[b25] Gu L, Baldocchi DD, Wofsy SC, Munger JW, Michalsky JJ, Urbanski SP, Boden TA (2003). Response of a deciduous forest to the Mount Pinatubo eruption: enhanced photosynthesis. Science.

[b26] Hungate BA, Van Groenigen K-J, Six J (2009). Assessing the effect of elevated carbon dioxide on soil carbon: a comparison of four meta-analyses. Global Change Biology.

[b27] IEA (2011). World Energy Outlook 2011.

[b28] Joos O, Hagedorn F, Heim A, Gilgen AK, Schmidt MWI, Siegwolf RTW, Buchmann N (2010). Summer drought reduces total and litter-derived soil CO_2_ effluxes in temperate grassland - clues from a ^13^C litter addition experiment. Biogeosciences.

[b29] Keith DW, Decarolis JF, Denjenberger DC, Lenschow DH, Malyshev SL, Pacala S, Rasch PJ (2004). The influence of large-scale wind power on global climate. Proceedings of the National Academy of Sciences.

[b30] King JS, Hanson PJ, Bernhardt E, Deangelis P, Norby RJ, Pregitzer KS (2004). A multiyear synthesis of soil respiration responses to elevated atmospheric CO_2_ from four forest FACE experiments. Global Change Biology.

[b31] Knapp AK, Fay PA, Blair JM (2002). Rainfall variability, carbon cycling, and plant species diversity in a mesic grassland. Science.

[b32] Lal R (2004). Soil carbon sequestration impacts on global climate change and food security. Science.

[b33] Lee H, EaG Schuur, Inglett KS, Lavoie M, Chanton JP (2012). The rate of permafrost carbon release under aerobic and anaerobic conditions and its potential effects on climate. Global Change Biology.

[b34] Luo Y, Wan S, Hui D, Wallace LL (2001). Acclimatization of soil respiration to warming in a tall grass prairie. Nature.

[b35] Ma S, Baldocchi DD, Xu L, Hehn T (2007). Inter-annual variability in carbon dioxide exchange of an oak/grass savanna and open grassland in California. Agricultural and Forest Meteorology.

[b36] Menzel A, Fabian P (1999). Growing season extended in Europe. Nature.

[b37] Mercado LM, Bellouin N, Sitch S, Boucher O, Huntingford C, Wild M, Cox PM (2009). Impact of changes in diffuse radiation on the global land carbon sink. Nature.

[b38] Millenium Ecosystem Assessment (2005). Ecosystems and Human Well-being: Synthesis.

[b39] Millstein D, Menon S (2011). Regional climate consequences of large-scale cool roof and photovoltaic array deployment. Environmental Research Letters.

[b40] Murata N, Takahashi S, Nishiyama Y, Allakhverdiev SI (2007). Photoinhibition of photosystem II under environmental stress. Biochimica et Biophysica Acta (BBA) - Bioenergetics.

[b41] Norby RJ, Warren JM, Iversen CM, Medlyn BE, Mcmurtrie RE (2010). CO_2_ enhancement of forest productivity constrained by limited nitrogen availability. Proceedings of the National Academy of Sciences.

[b42] Ostle NJ, Levy PE, Evans CD, Smith P (2009a). UK land use and soil carbon sequestration. Land Use Policy.

[b43] Ostle NJ, Smith P, Fisher R (2009b). Integrating plant–soil interactions into global carbon cycle models. Journal of Ecology.

[b44] Parton W, Silver WL, Burke IC (2007). Global-scale similarities in nitrogen release patterns during long-term decomposition. Science.

[b45] Pearce-Higgins JW, Stephen L, Douse A, Langston RHW (2012). Greater impacts of wind farms on bird populations during construction than subsequent operation: results of a multi-site and multi-species analysis. Journal of Applied Ecology.

[b46] Peng S, Piao S, Wang T, Sun J, Shen Z (2009). Temperature sensitivity of soil respiration in different ecosystems in China. Soil Biology and Biochemistry.

[b47] Pogson M, Hastings A, Smith P (2013). How does bioenergy compare with other land-based renewable energy sources globally?. GCB Bioenergy.

[b48] Reich PB, Tilman D, Craine J (2001). Do species and functional groups differ in acquisition and use of C, N and water under varying atmospheric CO_2_ and N availability regimes? A field test with 16 grassland species. New Phytologist.

[b49] REN21 (2012). Renewables 2012 Global Status Report,.

[b50] Sardans J, Rivas-Ubach A, Peñuelas J (2012). The C:N:P stoichiometry of organisms and ecosystems in a changing world: a review and perspectives. Perspectives in Plant Ecology, Evolution and Systematics.

[b51] Scherba A, Sailor DJ, Rosenstiel TN, Wamser CC (2011). Modeling impacts of roof reflectivity, integrated photovoltaic panels and green roof systems on sensible heat flux into the urban environment. Building and Environment.

[b52] Singh BK, Bardgett RD, Smith P, Reay DS (2010). Microorganisms and climate change: terrestrial feedbacks and mitigation options. Nature Reviews Microbiology.

[b53] Smith JU, Graves P, DR Nayak (2011). Carbon Implications of Windfarms Located on Peatlands – Update of the Scottish Government Carbon Calculator Tool.

[b54] Sulman BN, Desai AR, Saliendra NZ (2010). CO_2_ fluxes at northern fens and bogs have opposite responses to inter-annual fluctuations in water table. Geophysical Research Letters.

[b55] Swift RS (2001). Sequestration of carbon by soil. Soil Science.

[b56] Van Groenigen K-J, Six J, Hungate BA, De Graaff M-A, Van Breemen N, Van Kessel C (2006). Element interactions limit soil carbon storage. Proceedings of the National Academy of Sciences.

[b57] Wang C, Prinn RG (2010). Potential climatic impacts and reliability of very large-scale wind farms. Atmospheric Chemistry and Physics.

[b58] Wise RR, Olson AJ, Schrader SM, Sharkey TD (2004). Electron transport is the functional limitation of photosynthesis in field-grown Pima cotton plants at high temperature. Plant, Cell & Environment.

[b59] Wu C, Niu Z, Gao S (2010). Gross primary production estimation from MODIS data with vegetation index and photosynthetically active radiation in maize. Journal of Geophysical Research.

[b60] Zhou L, Tian Y, Baidya Roy S, Thorncroft C, Bosart LF, Hu Y (2012). Impacts of wind farms on land surface temperature. Nature Climate Change.

